# European Correspondence

**Published:** 1877-12

**Authors:** N. J. Nunn

**Affiliations:** London


### EUROPEAN CORRESPONDENCE.

London, October 9, 1877.

Since listening to the very able address delivered by Prof. Robert before the British Medical Association at Manchester of which I sent you a resume, I have been engaged in some efforts to adapt one of the more recent discoveries in antisepticism which, up to the present time, have been confined to the physiological laboratory—to economic purposes.

It is now an established fact, that an organic body may be kept without change for an extended period of time in contact with air which has been filtered through cotton—provided none of the seeds (so to speak) of decomposition were present at the time the body was placed in the filtered atmosphere. This curious fact depends upon the power possessed by cotton of filtering all organic matters from the atmosphere passed through it, and hence permitting nothing but pure gases to come in contact with the body enclosed within its protecting envelope.

From this it will be understood that decomposition appears to be, to a certain degree, dependent upon seeds (scientifically called spores) floating in the atmosphere, which, coming in contact with organic bodies, under favorable conditions, set up the changes which we call putrefaction.

The next point to be understood is, that a heat far less than that required to scorch cotton, will destroy the vitality of these seeds; indeed, for all practical purposes, the heat of boiling water has so far proved effectual. Every housekeeper knows that boiled milk keeps longer than unboiled, and every cook can tell us that heating meat, or "setting it," notably retards decomposition. This, again, is the oft-told tale of practical experience leading the van of science, and is the principle which underlies the preservation of food by "canning" or by "bottling;" in other words, by hermetically sealing, in which the heat employed destroys the germs, and then the ingress of the atmosphere is prevented by mechanical means; but the appliances are expensive, and much care, and sometimes mechanical skill, are needed to insure success. The least carelessness results in certain failure, and hence food preservation by the process of hermetical sealing, can never become of daily use in the household, the kitchen, the apothecary's laboratory, or the invalid's chamber.

The process which I desire to bring to the attention of your readers and of the public, is simplicity itself. Every housewife can practice it successfully. It needs no special apparatus; it requires no particular mechanical dexterity, and yet it will preserve the most delicate foods untainted, and keep easily putrescible fluids sweet for any reasonable length of time.

Suppose it is desired to preserve a pint of milk. Put it into a sufficiently large vessel to allow room for boiling. The material or shape of the vessel is of no practical importance, so far as the principle of preservation is concerned, but a glass bottle will be found useful and convenient, as permitting the process to be observed. If a glass bottle is employed, it would be well to have it annealed by slowly heating it to the temperature of boiling water, and then permitting it gradually to cool. The milk having been put into such a bottle, the mouth is to be covered with a ball of cotton, which is to be tied securely round the neck, so that no air can enter except through the cotton, and thus prepared the bottle is to be put into the oven, or otherwise heated, until the fluid boils; the heat is then withdrawn and the process is finished.

Now as long as that cotton is not disturbed the milk will remain perfectly sweet, but if the cotton is once removed, the process must be repeated or the contents of the bottle will spoil. If, therefore, small quantities are required at frequent intervals, it will be found more convenient to have a number of vessels, and to put into each only so much as will be needed at one time. It is quite possible, however, if the vessel is provided with a stopcock to withdraw such small quantities of fluid at a time as may be desired—the vacuum in the vessel being supplied with air filtered through the cotton. In a similar way any other organic fluid may be preserved.

The convenience and value of this simple process are, in my opinion, very great. It will permit of soups, broths, etc., being kept from day to day, from week to week, nay, even from month to month, if desired, with little or no trouble or expense, as far as the process is concerned, but with a positive saving of food, which, in small families particularly, might otherwise spoil and be thrown away.

If it is desirable to prepare the vessel so as to admit of transportation, it is only necessary to put a cork loosely into the neck of the bottle before the cotton is put on, the whole is then heated, and when it has cooled the cork is forced into the neck of the bottle by pressure on the outside of the cotton.

Keeping in view the principle that everything coming in contact with the fluid must be purified by a heat of 212 degrees Fahrenheit, and that all air permitted to enter must be made to pass through cotton; the stopping of the vessel may be accomplished in a variety of ways; but the plan just proposed will suit for domestic purposes, but, as being the simplest, another method will however be mentioned when the use of the principle in its commercial application comes to be discussed.

The employment of any of the preserving jars or cans at present in use will be much facilitated by the adoption of this process, as by putting the cover loosely in place, and surrouding it with cotton, the vessel can be left to cool before the cover is fastened down, and so the pressure inside and outside the can or jar being equalized, the tendency to leakage will be reduced to a minimum, but it must be borne in mind that in fastening the cover, the safest and best way is to do so without disturbing the ball of cotton.

Every nurse knows the difficulty of dealing with infants' food. This simple process solves the problem. A few vials each to hold food enough for a meal, a little cotton and a hot oven or a saucepan of boiling water arė the only utensils required, and then food sufficient for a week can be prepared with perfect certainty of being found perfectly sound at the end of that time.

Then for the sick-room, beef tea, broth, gruel, etc., can be prepared during the day, and be always ready at the moment required. What practitfoner will not recognize the immense convenience, if not value, of a simple process which will enable him with certainty, to have food ready for his patient at the very moment when the invalid's capricious fancy will admit of his taking nourishment which a few moments later he would reject, while by reducing to a minimum the immense waste by spoiling, it will so diminish the cost and trouble attending the preparation of the diet, as to allow of a greater variety of food and tempting luxuries being kept constantly on hand without any increase of expense. These reasons show how it will bring within the reach of the sick poor many luxuries which they are now unable to procure.

Here, also, it opens up a new field for the wealthy and philanthropic, who can thus at their leisure prepare various kinds of food and luxuries for the destitute sick, and, at convenient opportunities, send them wherever they may be needed.

Unless a more extended experience than I have had in the use of this process should develop difficulties which have not occurred to me, the application of this principle will prove of great advantage to the physician and to the apothecary. The former may prescribe infusions or decoctions, and the latter can keep them on hand, and supply them without being compelled to have recourse to the use of alcohol to preserve them, or to the questionable fluid extract, to furnish an unreliable substitute for them. Thus:

Into a tin can having a stopcock at the bottom put the materials required, close and cover the mouth of the can with cotton, as in the case of the milk already described, then let the whole be heated to boiling point, and preservation is complete. The contents may now be drawn off through the stopcock, at such intervals and in such quantities as required.

Next let me describe the plan I have adopted to preserve solids—such as pieces of meat—using, of course, the ordinary utensils to be found in every kitchen. Put the meat on a dish, put over it a clean towel to keep the cotton from the meat; pack raw cotton, evenly, all round on the edge of the dish; next put on a dish cover (in which there must be no holes) and see that there is cotton between the edge of the cover and the dish all round; next tie the cover down as tightly as possible, or else put on to the cover a good heavy weight; as a further precaution, put, if possible, more cotton round the outside of the joint between the cover and the dish; finally, heat the whole in the oven to 212 degrees Fahrenheit.

This method will also suit for fish, fowl, game, or any other sold food, and if smaller pieces are to be operated on, of course smaller vessels may be used, still remembering to cover with cotton, and then to heat to 212 degrees Fahrenheit.

If the object is to prepare cans, such as are commonly sold, the adaptation of this process will be found to be very simple. Having the can filled and the top soldered on, make a small hole in it to allow the escape of the air as it expands; into this hole loosely fit a peg of solder, cover the top with cotton, and screw it on in any convenient way; heat to 212 deg. F.; let the can cool, press the peg of solder tightly into the hole, remove the cotton, clip the peg close to the can, put over it a drop of solder, and the operation is complete. Other variations of the mechanical details have been tried but the one described seems to be the simplest.

This principle is also applicable to the evaporation of fluids, as in condensing milk and making extracts, because any solution of organic matter which has been boiled and not again exposed to unfiltered air, may be dried without decomposition by a current of air filtered through cotton; and the same thing is true with regard to the dessication of solids, animal as well as vegetable, at the ordinary temperature of the atmosphere. In many instances, the primary heating will prove almost an insurmountable objection, but it is quite possible that a rapid current of filtered air may dry the surface of the body so rapidly, and perhaps wash away the spores upon it, that decomposition would not set in before dessication would be completed. My experiments on this point are not sufficiently extended to warrant me in giving a decided opinion.

The use of cotton as a surgical dressing, is far from being a novelty in the South, although in the North and in Europe some surgeons claim no little credit for its introduction. It is quite possible that its abundance and convenience may have been the causes which influenced its employment, and that we, in common with the rest of the world were aware of the protective power it possessed. Be this as it may, its practical value was fully appreciated, as its frequent use in the treatment of burns, scalds, etc., will testify; still, it is worthy of a more careful, extended and scientific investigation. In combination with a spray of carbolic acid or some other suitable agent, it would, perhaps, prove to be a cheap and effectual substitute for more expensive and troublesome antiseptic dressings. It is quite possible that even the father of antiseptic surgery, Prof. Lister, might be induced to bring his large experimental experience to bear on its employment.

An interesting experiment which could be tried in some establishment where artificial ventilation is employed, would be to test the preservation of organic substances in apartments through which a constant stream of filtered air would be driven; in other words, into which the ingress of the atmosphere would be prevented by the outward rush of a current of filtered air. Should such an experiment prove successful it might be a valuable mode of ventilation in hospitals, dissecting rooms, etc., and even be deemed sufficiently useful to lead to its adoption in manufacturing establishments where putrescible substances are employed, such as meat-packers, dairies, etc.

Permit me to express the hope that these remarks will induce some experimenters, with better opportunities at their command than I have, to undertake the further investigation of this interesting subject; but, at the same time, let it not be forgotten to accord a full mead of praise to the discoverer of the air-filtering power of cotton—Prof. Tyndall.

N. J. Nunn. M.D.

